# Innovative Peptide-Based Plasmonic Optical Biosensor for the Determination of Cholesterol

**DOI:** 10.3390/bios14110551

**Published:** 2024-11-13

**Authors:** Ana Lia Bernardo, Anne Parra, Virginia Cebrián, Óscar Ahumada, Sergio Oddi, Enrico Dainese

**Affiliations:** 1 Biochemistry and Molecular Biology Unit, Department of Bioscience and Technology for Food, Agriculture and Environment, University of Teramo, Campus “Aurelio Saliceti” Via Renato Balzarini n. 1, 64100 Teramo, Italy; albernardoleonardi@unite.it; 2 Mecwins S.A., Ronda de Poniente, 15, 2°D, Tres Cantos, 28760 Madrid, Spain; aparra@mecwins.com (A.P.); vcebrian@mecwins.com (V.C.); oahumada@mecwins.com (Ó.A.); 3 Department of Veterinary Medicine, University of Teramo, Via Renato Balzarini n. 1, 64100 Teramo, Italy; 4 European Center for Brain Research (CERC), Santa Lucia Foundation I.R.C.C.S., Via del Fosso di Fiorano 64, 00143 Rome, Italy

**Keywords:** peptide, biosensor, cholesterol, localized surface plasmon resonance, dark-field microscopy

## Abstract

Plasmonic-based biosensors have gained prominence as potent optical biosensing platforms in both scientific and medical research, attributable to their enhanced sensitivity and precision in detecting biomolecular and chemical interactions. However, the detection of low molecular weight analytes with high sensitivity and specificity remains a complex and unresolved issue, posing significant limitations for the advancement of clinical diagnostic tools and medical device technologies. Notably, abnormal cholesterol levels are a well-established indicator of various pathological conditions; yet, the quantitative detection of the free form of cholesterol is complicated by its small molecular size, pronounced hydrophobicity, and the necessity for mediator molecules to achieve efficient sensing. In the present study, a novel strategy for cholesterol quantification was developed, leveraging a plasmonic optical readout in conjunction with a highly specific cholesterol-binding peptide (C-pept) as a biorecognition element, anchored on a functionalized silica substrate. The resulting biosensor exhibited an exceptionally low detection limit of 21.95 µM and demonstrated a linear response in the 10–200 µM range. This peptide-integrated plasmonic sensor introduces a novel one-step competitive method for cholesterol quantification, positioning itself as a highly sensitive biosensing modality for implementation within the AVAC platform, which operates using reflective dark-field microscopy.

## 1. Introduction

Cholesterol is a fundamental lipidic metabolite in the human body and a crucial component of all cell membranes. It plays a key role in maintaining cell membrane structure, function, and dynamics through its general effects on membrane proteins, thus supporting various essential biological processes [1,2,3]. Cholesterol is also the precursor of steroid hormones, vitamin D, bile acids, and brain myelin, and is thus, crucial in neural networks.

The accumulation of circulating cholesterol in the blood, where it is bound to lipoproteins, and the dysregulation of its homeostasis are associated with metabolic disorders and inflammatory activation of immune cells, contributing to cardiovascular diseases (CVDs), as well as neurodegenerative and neuroinflammatory disorders such as Parkinson and Alzheimer’s disease [4,5,6]. Abnormal cholesterol levels outside the normal range of 3.23–5.17 mM (125–200 mg/dL) in humans have been identified as a risk factor for atherosclerosis and CVD, among other illnesses [7,8,9]. However, little attention is paid to low cholesterol levels, which also can have serious health consequences, including an increased risk of depression, anxiety, and even suicidal tendencies [10]. Indeed, low serum levels of free cholesterol are associated with disorders like malabsorption syndromes, cancer, hyperthyroidism, and hemorrhagic stroke, particularly when cholesterol synthesis or absorption is impaired [11,12,13,14]. Therefore, the development of sensitive, accurate, and time-saving cholesterol biosensors is essential for monitoring cholesterol levels and enabling early disease diagnosis in clinical and medical applications.

Detection of small molecules such as cholesterol is challenging because of the inherent complexity involved in conventional sandwich assays utilizing antibody pairs [15]. Over the years, advances in biotechnology and nanotechnology have led to the design of a plethora of cholesterol biosensors mainly based on enzymatic detection by cholesterol oxidase (ChOx) or host–guest complex with β-cyclodextrin, resulting in colorimetric, fluorescent, or electrochemical devices [16,17,18,19,20,21,22,23]. Optical imaging methods such as plasmonic-based sensing are currently garnering attention, as they show significant advantages over conventional analytical methods, like enhanced sensitivity and specificity, higher signal-to-noise ratio, and the possibility of direct testing, overcoming the necessity of component or sample pretreatments and complicated and time-consuming procedures [24,25,26,27]. In this regard, optical biosensing is conducted by transduction of the optical signal generated when the ideal biomolecular interaction between the bioreceptor and analyte takes place [28].

Moreover, gold nanoparticles (GNPs) have been recognized as promising scaffolds in plasmon resonance platforms for the highly sensitive and selective detection of small molecules, biological targets, and molecular diagnosis [29,30,31,32]. GNPs exhibit distinctive physicochemical characteristics, including easily tunable surface chemistry due to their high surface-to-volume ratio, biocompatibility, and unique optoelectronic properties [32,33,34,35,36]. The potential application of GNPs is based on their ability to function as highly sensitive and selective transducers for binding events between analytes and recognition elements, leveraging their localized surface plasmon resonance (SPR) properties, making them reliable sensors [37]. The plasmon resonance phenomenon involves the excitation of surface electrons in a metal when irradiated with incident light at a specific wavelength, resulting in the collective coherent oscillation of the electronic charge. This process generates localized plasmon resonance, electromagnetic waves, and intense light scattering of the absorbed light [38,39,40,41,42].

Peptide-based biosensors have recently emerged as promising biorecognition elements due to their exceptional stability, chemical versatility, tunability, structural diversity, and high affinity for proteins [43]. For instance, specific peptide sequences exhibit higher affinity towards target analytes with enhanced conformational and chemical stability compared to proteins, thereby facilitating the development of sensitive biosensors for the recognition of biological targets or small molecules [44,45]. As previously mentioned, cholesterol modulates the activity of several membrane proteins through its interaction with the transmembrane domain of these proteins [1,46]. Consequently, in the context of lipid–protein interaction mechanisms, significant attention has been directed towards the cholesterol recognition/interaction amino acid consensus (CRAC) motif, as putative peptide sequences containing this motif are predicted to bind cholesterol within cellular membranes [47,48,49].

The integration of the superb properties of small peptides and GNPs within a single system thus provides an opportunity to achieve sensitive single-molecule plasmonic biosensing, addressing the limitations of current optical and colorimetric sensors for the detection of low molecular weight molecules [45,50,51,52].

In this study, we have devised an innovative strategy that exploits the scattering properties of GNPs’ SPR in the presence of a dielectric silicon-based substrate. GNPs functionalized with cholesterol molecules were used as optical markers, and the light scattered by GNPs immobilized on the surface was optically detected and monitored using AVAC technology, a dark-field microscopy-based method [53,54,55,56].

We exploited the affinity and conformational selectivity of a CRAC motif-based peptide sequence (ATVLNYYVWRDNS) selected from the murine peripheral-type benzodiazepine receptor (PBR), hereafter referred to as C-pept [57], to develop a novel plasmonic biosensor for the quantitative detection of cholesterol through a competitive assay approach leveraging AVAC technology. This strategy enables rapid, high-throughput screening and presents potential applications in future point-of-care biosensors. One of the main challenges of this study was adapting sandwich immunoassay technology to detect a low molecular weight molecule such as cholesterol. The system presented herein is the first to employ a cholesterol-binding peptide as a biorecognition element in a plasmonic biosensor based on a competitive assay for the quantification of free cholesterol.

## 2. Materials and Equipment

### 2.1. Materials

One-side polished silicon wafers were purchased from Si-Mat-Silicon Materials e.K. (Kaufering, Germany). Peptide sequences, herein called C-pept (ATVLNYYVWRDNS) and Pept-4 (ATVLNYYVWHDNS), were synthesized by ProteoGenix S.A. (Schiltigheim, France). Cholesterol-PEG-NH2 was purchased from Abbexa Ltd. (Cambridge, UK). N-(3-dimethylaminopropyl)-N′-ethylcarbodiimide hydrochloride (EDC), N-hydroxysulfosuccinimide sodium salt (Sulfo-NHS), cholesterol powder, 4-morpholineethanesulfonic acid MES low moisture content ≥ 99%, (3-aminopropyl)triethoxysilane (APTES), glutaraldehyde solution 50 wt% in H2O, poly(ethylene glycol) bis(amine) Mw: 20000 (PEG-diamine 20 kDa), poly(ethylene glycol)diamine Mn: 3000 (PEG-diamine 3 kDa), Tween20, and 1X phosphate-buffered saline (PBS) tablets were purchased from Sigma-Aldrich S.L (Madrid, Spain). All reagents were of analytical grade. All solutions and buffers were prepared using ultrapure milli-Q water. Commercial gold nanoparticles (GNPs) with carboxyl polymer coating were obtained from Nanopartz (Salt Lake City, UT, USA) (GNPs-C11-100-TC-DICH-50-1) with a standard diameter of 100 nm. FlexWell one-side adhesive 16-well incubation chambers made of clear silicone (6.8 × 6.8 × 3.2 mm depth) were purchased from Grace Bio-Labs Inc. (Bend, OR, USA). All aqueous solutions were prepared in ultra-pure milli-Q water with a resistivity of 18.2 MΩ cm (at 25 °C).

### 2.2. Equipment

Optical detection, analysis, and quantification of the plasmonic GNPs were performed using an AVAC platform (Mecwins S.A, Madrid, Spain) with the corresponding analysis software, and a Stuart rotator SB2 (VWR International, Barcelona Spain), ultrasound cleaning bath USC100T (VWR International, Barcelona, Spain), vortex mixer Reaxtop (VWR International, Barcelona, Spain) and microcentrifuge PrismR (Labnet Edison, Madrid, Spain) were used for the functionalization of GNPs. A UV/Vis spectrophotometer Nanodrop2000 (Thermo Scientific S.L Madrid, Spain) was used for the quantification of GNPs. The KSV CAM 200 optical contact angle meter (KSV Instruments Ltd., Helsinki, Finland) was used for the measurements of the water contact angle (WCA) of liquid droplets on the functionalized surfaces, and images and analysis were processed with CAM200 software (ver. 4.01) in static mode.

## 3. Methods and Experimental Part

### 3.1. Silica Surface Functionalization with Glutaraldehyde Chemistry for Peptide Immobilization

Silanization of the silicon (Si) wafers was conducted via chemical vapor deposition (CVD). The silicon substrate was amine-functionalized by reaction with (3-aminopropyl)triethoxysilane (APTES), and the Si-APTES surfaces were stored under vacuum at 4 °C until their use. The Si-APTES wafers were cut into slides with a dimension of 27 × 77 mm for the 16-well experiments.

The Si-APTES slides were treated with a 5% glutaraldehyde (GA) solution in 1X PBS on a rocker for 1 h at room temperature for the purpose of surface functionalization. The slides were rinsed with PBS and milli-Q water twice for 5 min each, avoiding drying of the surface between washes. Then, the slides were dried under nitrogen flux, and 16-well adhesive chambers were carefully roll-pressed on their functionalized side.

Afterwards, the slides were incubated with 150 µL of 10 µg/mL (1%) of PEG linker in PBS for 30 min at 37 °C. PEG diamine was selected as a spacer between the surface and the peptide to reduce the possible false positives and create an oriented position of the peptide.

In the meanwhile, solutions of EDC and Sulfo-NHS were prepared in an aqueous solution in acidic conditions. The stock solution of C-pept in milli-Q water was diluted to 80 µg/mL in MES with pH 5.5.

Finally, the covalent coupling of C-pept on the pegylated substrate was achieved by using the EDC/NHS carbodiimide method. The slides were rinsed with PBS and immediately incubated with 150 µL/well of the vortexed peptide solution pre-mixed with a 1:1 solution of EDC/NHS with 80 µg/mL of C-pept for 1 h at 37 °C on static. Prior to incubation of the GNPs for the bioassay, the surface was rinsed with PBS to remove unreacted peptide molecules.

### 3.2. Functionalization of Gold Nanoparticles (GNPs)

Commercial carboxylate gold nanoparticles (GNPs) obtained from Nanopartz with a standard diameter of 100 nm were selected for the experiments conducted. The concentration of the GNP stock in milli-Q water was calculated using Nanodrop, measuring its absorbance at 569 nm (SPR peak) to obtain the necessary volume to be added for its functionalization at a final concentration of 192.31 µg/mL. See Table 1.

Surface functionalization of GNPs was conducted via carbodiimide coupling by the terminal carboxylic acid groups. Then, in a low-binding Eppendorf tube, the appropriate amounts of Chol_PEG- NH_2_ and GNPs for the preparation of solutions at 100 µg/mL in PBS and 250 µg/mL in milli-Q water, respectively, were mixed with 1 mL of an aqueous solution in acidic conditions. Before any step of the GNP functionalization, the solution was sonicated to ensure homogenization.

To this mixed solution, 100 µL of each coupling reagent (EDC and NHS-Sulfo solutions) at a concentration ratio 1:1 in acidic conditions were immediately incorporated. Finally, the mixture was incubated for 1 h on a rotator at RT.

Thereafter, unreacted Chol_PEG-NH_2_ molecules were removed by centrifugation and washing in PBST (PBS 0.05% Tween) four times at 17,000× *g* and 4 °C for 5 min each in a microcentrifuge. Before each centrifugation step, GNPs were sonicated and further vortexed to avoid aggregation. Functionalized GNPs were stored in storage buffer solution at 4 °C until their use.

Before each experiment, the GNP concentration was determined by measuring the absorbance at around 569 nm with a NanoDrop spectrophotometer.

### 3.3. Competitive Plasmonic Bioassay for Cholesterol Determination

Before the determination of cholesterol, a cholesterol solution in ethanol with a concentration of 10 mM was prepared and stored at 4 °C until its use. After the washing steps, each well of the fully assembled Si/APTES/GA/PEG/C-pept substrate was incubated with 150 µL of 5 µg/mL GNPs-Chol in PBST for 1 h at 37 °C. The competitive assays were performed by adding increasing concentrations of free cholesterol to the bioassay buffer media in the presence of functionalized GNPs.

To verify the selectivity of the C-pept in the developed cholesterol plasmonic biosensor system and the sensitivity of the method, a modified peptide sequence lacking affinity for cholesterol was investigated under the same conditions, immobilizing the bioreceptor as explained in Section 3.1.

### 3.4. AVAC Analyzer

Analysis and quantification of surface-bound GNPs was performed with the AVAC platform, patented by Mecwins S.A. (US11519843B2 and US11519856B2). The AVAC platform represents an innovative approach to biomarker detection and quantification, using digital counting of plasmonic gold nanoparticles imaged with reflective dark-field microscopy. Characterization and classification of the particles seen in the dark-field images is achieved by analyzing the relative brightness and color of each particle. These two parameters can be represented in a two-dimensional histogram (Figure S1A), allowing to clearly distinguish different particle populations. Primarily, GNPs can be discriminated from other particles (chemical residues etc.), and, in addition, monomers, dimers, trimers, and clusters of GNPs can be distinguished, allowing to assign each particle found in the dark-field images to one of these categories (Figure S1B). For the quantification of GNPs, only the monomer population is taken into account. From the number of monomers, the amount of the analyte in the sample can be calculated [55,56].

### 3.5. Surface Characterization

Contact angle measurements were performed in a KSV CAM 200 optical contact angle meter connected to a computer supported with CAM200 software to process the images and analyze the WCA measurements. The volume used in the study was 1 μL of milli-Q water. After each step of surface modification, the values of the angle (*n* = 3) were obtained.

## 4. Results and Discussion

In this study, we aimed to develop an innovative method for detecting free cholesterol (Chol) using C-pept (Figure S2A), a 13-amino acid synthetic peptide previously characterized as a cholesterol-binding motif [57]. C-pept was chosen as the biorecognition element for an optical biosensor based on plasmonic gold nanoparticles (GNPs) and localized dark-field spectrophotometry. The effectiveness of plasmonic biosensing for the sensitive and direct monitoring of biomolecular interactions in in vitro assays is well-established in the scientific literature. Plasmonic biosensor prototypes are often designed around the antigen–antibody recognition event, either in label-free (direct) or sandwich (GNP-assisted) formats, which have shown significant promise as medical devices for the specific and sensitive detection of disease biomarkers in point-of-care applications. In this work, we extend the applicability of the conventional plasmonic biosensor by utilizing it as a sensitive biosensor for free cholesterol detection, employing the cholesterol-binding peptide C-pept in a competitive assay format.

### 4.1. Functionalization of Silicon Surface with C-Pept as the Recognition Element

In the context of plasmonic biosensors, the use of dielectric materials such as silica provides an ideal platform substrate, enhancing the sensitivity, stability, and functionalization capabilities [58,59]. The surface functionalization protocol for silicon was adapted and optimized considering the hydrophobicity, small size, and alpha-helical structure of C-pept, ensuring appropriate orientation and stability to enable effective interaction with cholesterol molecules.

**Scheme 1:**
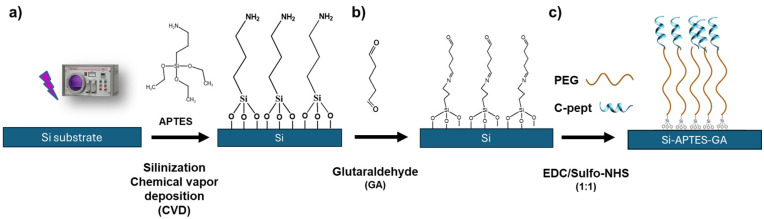
Schematic representation of surface modification steps involved in the construction of the cholesterol biosensor. (**a**) Surface activation and APTES deposition via CVD, (**b**) incorporation of GA crosslinker and (**c**) immobilization of the biorecognition element C-pept.

The surface functionalization strategy is illustrated in Scheme 1. Silicon wafers were silanized using CVD, activating the surfaces and forming a smooth monolayer of APTES [59]. The resulting amine-functionalized surface was sectioned into slides and incubated in a 5% GA solution for 1 h at room temperature. GA is widely employed as a crosslinker, enabling spontaneous covalent bonding to amino groups of biomolecules, such as antibodies [58,60]. In this study, we further optimized the platform design by introducing PEG diamine moieties as linkers. This strategy provided the necessary spacing for accurate C-pept/Chol biorecognition and ensured the vertical orientation and stability of C-pept, which was conjugated via the widely used EDC/NHS chemistry.

The sequential steps comprised a full wet-chemistry protocol where the crosslinking PEG moieties and further covalent immobilization of C-pept were performed directly on each well chamber. Employing this method, we were able not only to assess the reproducibility between different independent replicates, but also the accuracy of the functionalization steps, ensuring the correct deposition and homogeneity of the required reagents within the wells.

Additionally, water contact angle measurements were performed to characterize the sequential chemical modifications of silicon surfaces, from initial Si activation to final functionalization. Variations in the contact angle values of water droplets on the modified surface correlated with the introduction of hydrophilic and hydrophobic groups, reflecting the chemical changes induced by the incorporated elements during the functionalization process.

Analysis of the contact angle measurements (Table 2) confirmed variations in the hydrophilicity properties of the silicon surface and the subsequent incorporation of functional moieties during the biofunctionalization process. Notably, the water contact angle (WCA) exhibited a significant increase from 67 ± 3° to 78 ± 3° on the PEGylated substrate upon the incorporation of C-pept. This change is attributed to the hydrophobic nature of the selected peptide and the presence of charged molecules on the surface.

### 4.2. GNPs Functionalization with Cholesterol

One of the most commonly employed methods for GNP biofunctionalization involves the direct covalent bonding of thiol-containing biomolecules to its surface via strong and stable sulfide bonds. Alternatively, EDC/NHS coupling chemistry is frequently used to conjugate bioreceptors through their amino or carboxyl groups and to incorporate linkers or adapter molecules such as avidin/biotin [35,61]. Moreover, to ensure proper orientation of the biomolecule, reduce nonspecific interactions, and enhance GNP stability and biocompatibility, bifunctional linkers and/or polymeric ligands are typically used as coating agents.

In this study, we aimed to covalently functionalize GNPs with cholesterol molecules. Cholesterol is known to be highly hydrophobic and insoluble in aqueous media unless organic solvents or detergents such as Triton X-100 or Tween 20 are present, and its only functional moiety is a hydroxyl group [62]. To address this, we employed a strategy to anchor PEGylated cholesterol molecules onto the GNP surface [63]. By using PEG as a linker, we mitigated potential steric hindrance, detachment, and incorrect orientation of cholesterol during its interaction with the peptide on the Si substrate, thereby reducing the cost and time required for the conjugation reaction.

**Scheme 2:**
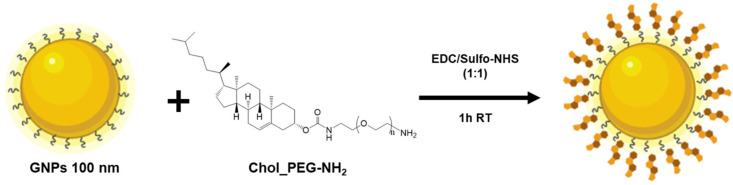
Synthetic route of the surface functionalization of GNPs with Cholesterol_PEG_NH_2_.

GNPs-COOH with an average size of 100 nm in milli-Q water were functionalized through carbodiimide coupling with Chol_PEG molecules, as illustrated in Scheme 2. Stock solutions of Chol_PEG-NH_2_ and GNPs were diluted to 100 µg/mL and 250 µg/mL, respectively, in 100 µL of PBS and milli-Q water, and mixed with 100 µL of EDC/NHS (1:1) coupling agents in 1000 µL of an aqueous solution under acidic conditions.

To confirm the correct conjugation, concentration, and agglomeration state of the GNPs, shifts on the SPR peak and signal intensity were studied by measuring the absorbance at 569 nm before and after the functionalization process using a UV-visible Nanodrop spectrophotometer. The functionalized GNPs (GNPs-Chol) were stored in PBST (0.05%) at 4 °C and remained stable without presenting significant aggregation for 3 days.

### 4.3. Peptide-Based Plasmonic Competitive Assay for Cholesterol Quantification

**Scheme 3:**
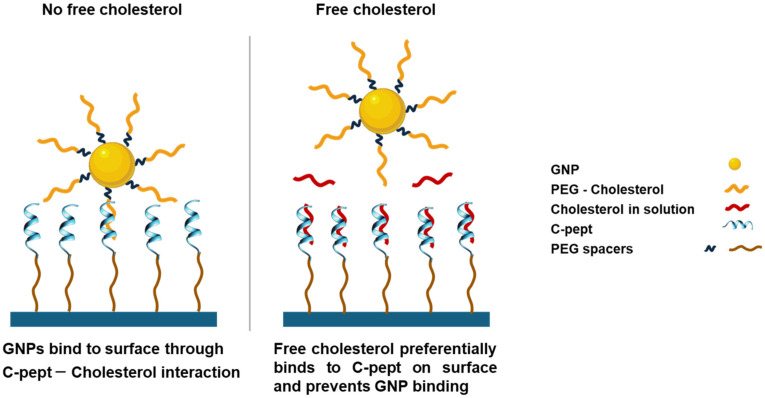
Schematic representation of the competitive assay proposed in this work. The presence of free cholesterol in solution competes with cholesterol-functionalized GNPs for binding to C-pept, the cholesterol sensing element, immobilized on the sensor surface.

In this work, the main challenge and limitation for the detection of free cholesterol molecules via sandwich assays derive from their small size and amphiphilic character. In addition, the precise orientation and conformation on an alpha-helix secondary structure of the peptide is crucial for the particular interaction with cholesterol molecules through its apolar-aromatic-basic branching sequence. Hence, to overcome this obstacle and achieve the detection and quantification of cholesterol by C-pept, a competitive assay strategy was followed, as depicted in Scheme 3.

In the proposed competitive assay for cholesterol detection, cholesterol molecules were immobilized onto beads, hereafter referred to as GNP-Chol, which served as the immobilized component. A peptide with high affinity for recognizing and binding cholesterol (C-pept) was immobilized onto the dielectric Si substrate. In this assay, the peptide can bind either to the cholesterol immobilized on the beads or to the free cholesterol present in the sample. Thus, free cholesterol in the sample competes with GNP-Chol for binding to the peptide. GNP-Chol was incubated in a 16-well chamber placed on the Si-C-pept surface. After the incubation period, unbound components were removed, and the number of GNPs bound to the peptide-functionalized surface was quantified. The results showed that the amount of GNPs bound was inversely proportional to the concentration of cholesterol in the sample, indicating that higher cholesterol levels result in fewer GNPs binding, thereby enabling the quantitative detection of cholesterol. Images of GNPs were captured under various conditions using an AVAC reader to quantify the GNPs immobilized by their interaction with C-pept. The plasmonic biosensor was thus designed to evaluate the competition between GNP-Chol and free cholesterol in solution as detected by C-pept.

**Figure 1:**
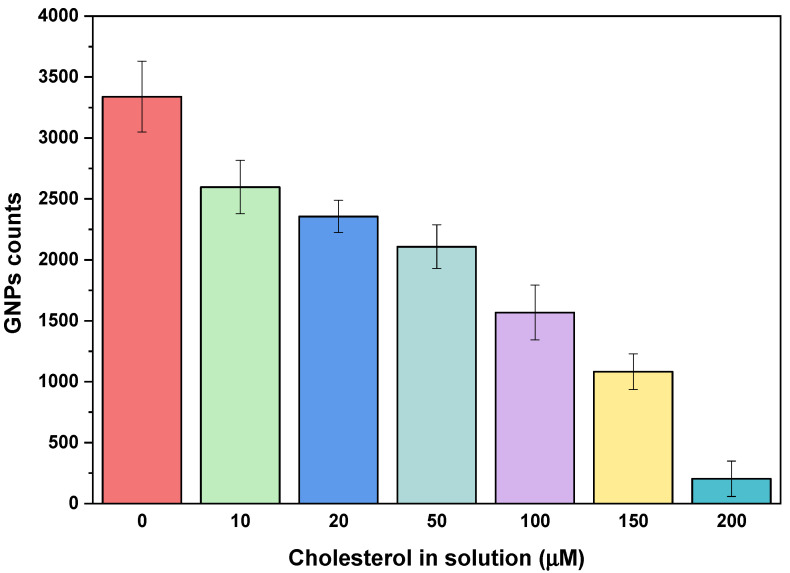
GNP-Chol counts of the cholesterol quantification bioassay conducted by the competitive interaction of GNPs functionalized with cholesterol and free cholesterol in a PBST (0.05%) solution. All data reported are presented as the mean ± standard deviation (SD) (*n* ≥ 3).

The sensitivity of the biosensing assay was assessed by exposing a fixed concentration of GNP-Chol to various concentrations of free cholesterol, ranging from 10 to 200 µM, in buffer media. It was observed that increasing concentrations of free cholesterol resulted in a reduction in the number of GNPs immobilized on the surface, as shown in Figure 1. The interaction of C-pept with GNP-Chol led to GNP immobilization on the surface; however, the presence of free cholesterol in the sample inhibited or displaced this interaction, likely due to steric and more accessible interactions between the small molecules. Thus, a competitive assay for free cholesterol quantification in solution was performed. The quantitative analysis revealed a linear response (R^2^ = 0.990) over the cholesterol concentration range of 10–200 µM, with a limit of detection (LOD) of 21.95 µM. The LOD was calculated using the equation LOD = 3σ/b, where σ is the standard deviation of the counts, and b is the slope (counts per concentration) (Figure S3). Furthermore, strong competition was observed when the system was exposed to 200 µM of free cholesterol, which may indicate the upper detection limit and saturation of the proposed system.

These results demonstrate the capability of C-pept to bind cholesterol molecules in aqueous media, suggesting its potential application as a sensitive biosensor for cholesterol quantification in small sample volumes.

The coefficient of variation (CV), calculated as the ratio of the standard deviation to the mean, for three independent experiments at each cholesterol concentration, ranging from 10 µM to 200 µM, was between 5.6% and 14.4%, confirming the sensitivity and reproducibility of the proposed method.

Subsequently, to validate the capability of the Si-APTES-GA-PEG-C-pept system to detect cholesterol, experiments were performed under the same conditions without peptide immobilization on the substrate, serving as a negative control.

**Figure 2:**
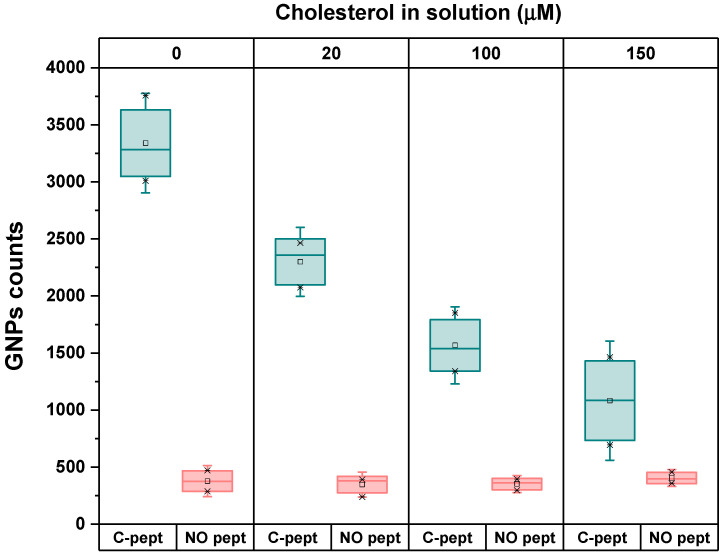
Evaluation of the sensitivity of the system. Chart-box plot of the counts of GNPs after interaction with the functionalized Si substrate in the presence (green box) and in absence (red box) of C-pept as biorecognition element. Effect of different concentrations of free cholesterol in solution when incubating GNP-Chol-PEG for 1 h at 37 °C in PBST. All data reported are presented as the mean ± standard deviation (*n* ≥ 3).

The results presented in Figure 2 demonstrate the recognition capability and high sensitivity of C-pept in the system for detecting cholesterol. The absence of a biorecognition element suggests that PEG moieties on the Si substrate are exposed, which may lead to nonspecific binding of GNPs through direct adsorption, crosslinking of functional groups, or aggregation. It is noteworthy that the signal of GNPs attached to the surface in the absence of cholesterol (control condition) was found to be eight-fold higher in the presence of the peptide. Furthermore, no significant changes in the GNP signal were observed on the control surface in the presence of free cholesterol at varying concentrations, remaining constant throughout the titration assay. Conversely, the presence of C-pept facilitated the correct interaction with cholesterol, as previously demonstrated. These findings validate the detection method using the proposed plasmonic system, confirming that GNP attachment resulted from the successful interaction and binding of the cholesterol-C-pept pair rather than from nonspecific binding.

### 4.4. Selection of the System Configuration

Conventional methods for immobilizing proteins or other bioreceptors on silicon surfaces typically employ APTES and GA as crosslinking agents. However, the APTES-GA method has limitations due to the use of GA, which contains two functional groups capable of binding nonspecifically to proteins, leading to irregular binding when the surface is not blocked. Additional spacers can be incorporated to mitigate this issue.

In this context, the correct orientation of C-pept is critical for its interaction and binding with cholesterol molecules, as defined by its putative cholesterol-binding motif. Furthermore, not only the orientation but also the conformational folding of C-pept can be affected by environmental and buffer media conditions.

Therefore, C-pept was immobilized on the surface via covalent linkage through its carboxy-terminal group to the amino groups of the PEG spacer. The effectiveness of its interaction with GNPs-PEG_Chol was evaluated using PEGs of different molecular weights (Mws). The results indicated that a higher Mw for the chosen spacer reduced steric hindrance, increased detection sensitivity, and minimized nonspecific signals in the proposed system [64].

**Figure 3:**
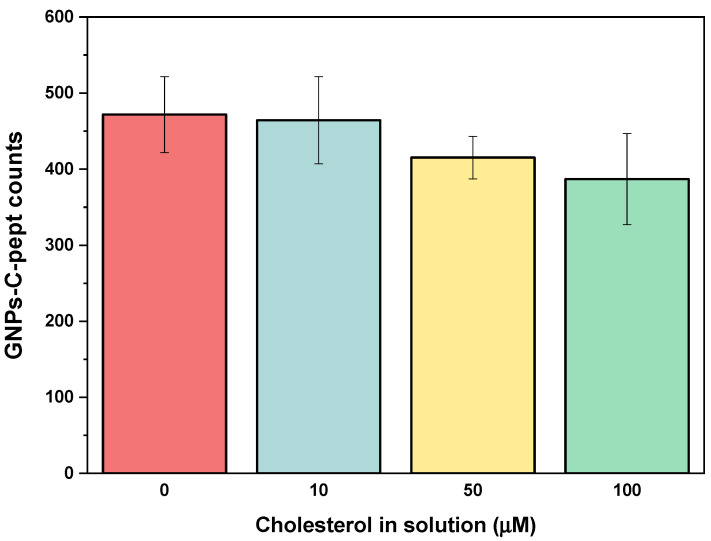
Quantification of free cholesterol in solution in the competitive bioassay implementing the system based on GNPs-PEG-C-pept and Chol_PEG immobilized on the Si-APTES-GA substrate at a fixed concentration. All data reported are presented as the mean ± standard deviation (*n* ≥ 3).

To further investigate the optimal configuration and functionality of the system, additional experiments were conducted using the inverted configuration, where PEG-Chol molecules were immobilized on the Si-APTES-GA substrate and GNPs functionalized with C-pept were utilized. Under these conditions, the competitive bioassay for free cholesterol in solution exhibited a decreasing trend, as expected from previous findings (Figure 3). Nevertheless, while the results confirmed the biorecognition and binding of C-pept to cholesterol, the interaction and specificity were lower when using this reversed configuration compared to the originally proposed biosensor strategy. The reduced number of GNPs interacting could be attributed to the denaturation of C-pept, affecting its proper alpha-helical conformation during the GNP functionalization process.

### 4.5. Specificity of the Biorecognition Peptide Element

We further evaluated the specificity of the plasmonic platform by selecting a non-binding cholesterol peptide. The peptide sequence chosen, designated as Pept-4 (ATVLNYYVWHDNS), was modified by altering one of the putative amino acids responsible for cholesterol binding within the CRAC motif. The sequence mutation resulted in a change in the peptide’s structure, destabilizing the ideal alpha-helix structure required for the CRAC residues to interact correctly on binding cholesterol (Figure S2B).

The protocol for surface functionalization and bioreceptor immobilization was carried out under the same conditions described in Section 3.2 for C-pept.

**Figure 4:**
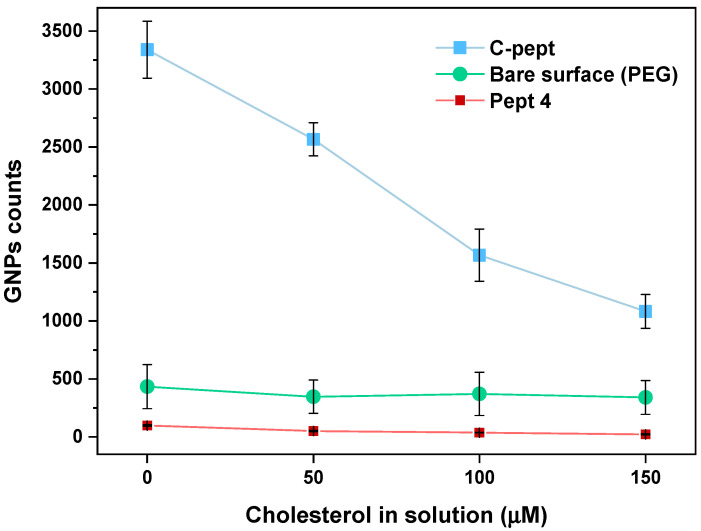
Specificity of the competitive bioassay of GNPs-Chol interacting with C-pept (blue), bare surface (green), and Pept 4 as negative control (red) in the presence of increasing concentrations of free cholesterol in PBST solution. All experiments were conducted under the same conditions (*n* ≥ 3).

As shown in Figure 4, the new sequence Pept-4, used as a negative control, exhibited a negligible interaction response with cholesterol. In contrast to the C-pept response, minimal plasmonic signals from GNPs were observed on the surface, both in the presence and absence of cholesterol as an interference. The resulting GNP binding was significantly lower than when no biorecognition element was present. This effect is directly attributed to minimal nonspecific binding of GNPs-Chol to the free terminal groups of PEG or GA, as no blocking agent was used. These findings confirm successful surface modification and verify that the observed results were indeed due to the specific molecular recognition between cholesterol and C-pept.

## 5. Conclusions

A novel plasmonic-optical biosensor based on a competitive assay for the detection of cholesterol has been successfully developed using C-pept, a cholesterol-binding peptide, as the biorecognition element. The biosensor employs a one-step competitive cholesterol bioassay system, involving the selective interaction of C-pept with cholesterol molecules immobilized on GNPs, which serve as optical markers. The AVAC platform facilitates the quantification of GNPs functionalized with PEG-Chol motifs bound to C-pept, competing with free cholesterol in buffer media, resulting in a calibration curve. This bioassay exhibits a highly sensitive detection limit of 21.95 µM, establishing it as an advanced, high-throughput tool for the quantification of free cholesterol that will presumably be valuable in clinical diagnostics, enabling early detection and monitoring of disorders linked to low free cholesterol. In conclusion, this peptide-based plasmonic approach provides novel insights for detecting lipidic, low molecular weight targets and metabolites, advancing the field of biosensing technology.

## Figures and Tables

**Table 1:** GNP stock solution properties supplied by Nanopartz (www.nanopartz.com) accessed on 4 October 2024.

**Diameter (nm)**	**100**
**Peak SPR Wavelength (nm)**	569
**NPS/ml**	2.48 × 10 ^11^
**Molarity (pM)**	4.10 × 10 ^2^
**Moles**	4.14 × 10 ^−13^
**Molar Ext. (M^−1^ cm^−1^)**	1.21 × 10 ^11^
**Absorption Molar Ext. (M^−1^ cm^−1^)**	5.82 × 10 ^10^
**Scattering Molar Ext. (M^−1^ cm^−1^)**	6.27 × 10 ^10^
**Size Dispersity %PDI**	<4%
**Size Accuracy (+/− nm)**	2

**Table 2:** Water contact angle values of the different functionalized surfaces.

Surface Modification	WCA (°)
Si	39 ± 2
Si-APTES	59 ± 1
PEG (linker)	67 ± 3
PEG-C-pept	78 ± 3
PEG-C-pept/GNPs-Chol	83 ± 5

## Data Availability

Data will be made available under request.

## Supplementary Materials

The following supporting information can be downloaded at: https://www.mdpi.com/article/10.3390/bios14110551/s1. Figure S1: (A) A two-dimensional histogram in which, for each particle, its relative brightness is represented on the horizontal axis and its relative red color component on the vertical axis. The number of particles with a certain combination of brightness and color is given using a color-coded scale, from none (white) or few (blue, green), to many (yellow, red). The populations of particles corresponding to monomers, dimers, trimers, and clusters of GNPs, and to residues (not GNPs), are indicated. (B) Dark-field microscopy image in which each particle has been assigned to a population according to the classification shown in the two-dimensional histogram. Inset: Transmission electron microscopy (TEM) image showing individual nanoparticles (monomers). Figure S2: 3D predicted structures of C-pept (A) and Pept 4 (B) obtained with Alphafold2 (https://alphafold.ebi.ac.uk) and PEP-FOLD3 (https://mobyle.rpbs.univ-paris-diderot.fr accessed on 3 April 2024) (Lamiable et al., 2016) [65] and best binding pose for cholesterol molecule determined by docking analysis with Autodock Vina software (https://vina.scripps.edu). Visualized with Chimera 13.50.53 (https://www.cgl.ucsf.edu/chimera accessed on 3 April 2024). Figure S3: Calibration curve of the competitive bioassay for the system Si-GA-APTES-C-pept for the quantification of free cholesterol in solution (PBST). All data reported are presented as the mean of n ≥ 3.
