# Supplementary material for: Innovative Peptide-Based Plasmonic Optical Biosensor for the Determination of Cholesterol
Source: Biosensors (Basel). 2024 Nov 13;14(11):551. doi: 10.3390/bios14110551 (PMC11592131; doi:10.3390/bios14110551)
Supplement: Supplementary file 1 [file biosensors-14-00551-s001.zip › biosensors-3276428-supplementary.pdf]

biosensors

MDPI

Supplementary Materials

Innovative Peptide-Based Plasmonic Optical Biosensor for the Determination of Cholesterol

Ana Lia Bernardo [1], Anne Parra [2], Virginia Cebrián [2], Óscar Ahumada [2,#], Sergio Oddi [3,4,*,#] and Enrico Dainese [1,*,#]

[1] Biochemistry and Molecular Biology Unit, Department of Bioscience and Technology for Food, Agriculture and Environment—University of Teramo, Campus "Aurelio Saliceti" Via Renato Balzarini n. 1, 64100 Teramo, Italy; albernardoleonardi@unite.it ((A.L.B); edainese@unite.it (E.D.)

[2] Mecwins S.A., Ronda de Poniente, 15, 2°D, Tres Cantos, 28760 Madrid, Spain; aparra@mecwins.com (A.P.); vcebrian@mecwins.com (V.C.); oahumada@mecwins.com (Ó.A.)

[3] Department of Veterinary Medicine—University of Teramo, Via Renato Balzarini n. 1, 64100 Teramo, Italy; soddi@unite.it (S.O.)

[4] European Center for Brain Research (CERC), Santa Lucia Foundation I.R.C.C.S., Via del Fosso di Fiorano 64, 00143 Rome, Italy; soddi@unite.it (S.O.)

* Correspondence: soddi@unite.it (S.O.); edainese@unite.it (E.D.)

\# These authors contributed as co-seniors to this work.

GNP detection and quantification

The detection, characterization, and quantification of GNPs is carried out with AVAC technology (patents US11519843B2 and US11519856B2). Spatially and spectrally resolved images are obtained with dark-field microspectrophotometry and subsequently analyzed. The analysis consists of correcting the images to reduce noise, localizing particles in the images, characterizing each particle with regard to its brightness and color, and classifying the particles. The brightness and a color component of each particle can be represented in a two-dimensional histogram (Fig S1A). Within this two-dimensional histogram, different populations can be distinguished, corresponding to monomers, dimers, trimers, and clusters of GNPs, and to residues (not GNPs), allowing to classify each particle that has been detected. Fig S1B shows an example of a dark-field microscopy image in which each particle has been assigned to a specific particle population. For the quantification of GNPs, only monomers are taken into consideration.

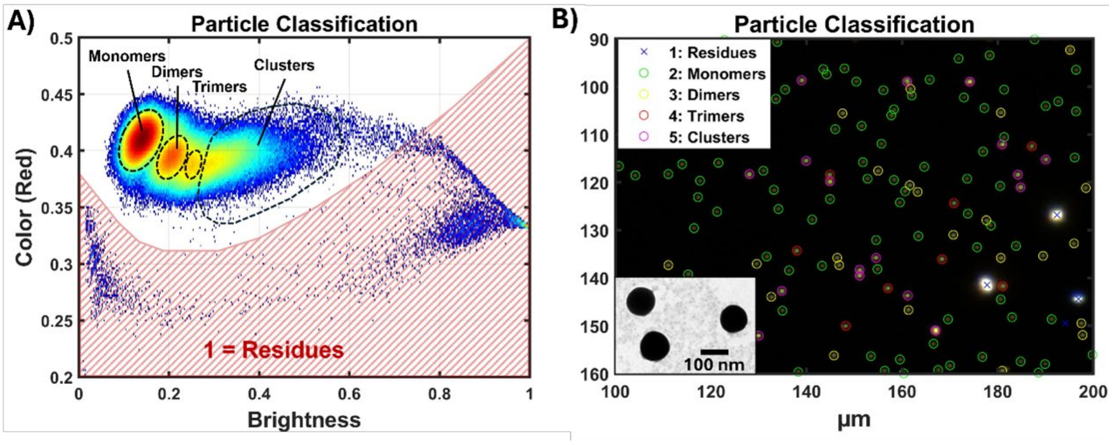

Figure S1. (**A**) A two-dimensional histogram in which, for each particle, its relative brightness is represented on the horizontal axis and its relative red color component on the vertical axis. The number of particles with a certain combination of brightness and color is given using a color-coded scale, from none (white) or few (blue, green) to many (yellow, red). The populations of particles corresponding to monomers, dimers, trimers, and clusters of GNPs, and to residues (not GNPs) are indicated. (**B**) Dark-field microscopy image in which each particle has been assigned to a population

according to the classification shown in the two-dimensional histogram. Inset: Transmission electron microscopy (TEM) image showing individual nanoparticles (monomers).

Study of C-pept used as a building block molecule for the biosensor design

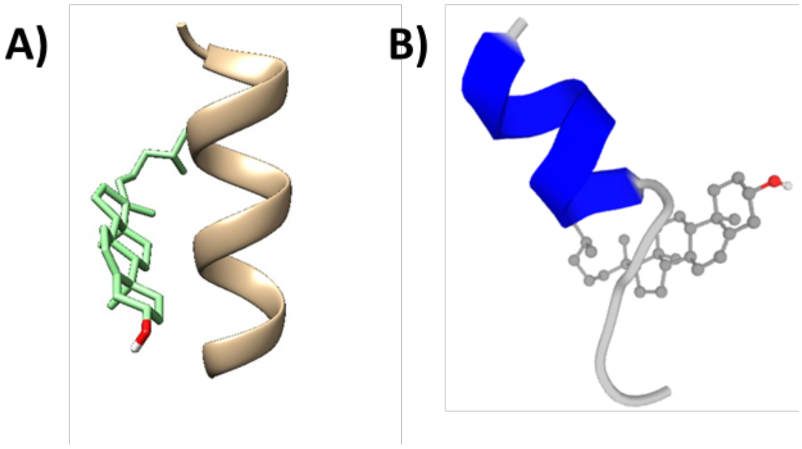

Figure S2. 3D predicted structures of C-pept (**A**) and Pept 4 (**B**) obtained with Alphafold2 (https://alphafold.ebi.ac.uk) and PEP-FOLD3 (https://mobyle.rpbs.univ-paris-diderot.fr accessed on 3 April 2024) (Lamiable et al., 2016) [65] and best binding pose for cholesterol molecule determined by docking analysis with Autodock Vina software (https://vina.scripps.edu). Visualized with Chimera 13.50.53 (https://www.cgl.ucsf.edu/chimera accessed on 3 April 2024).

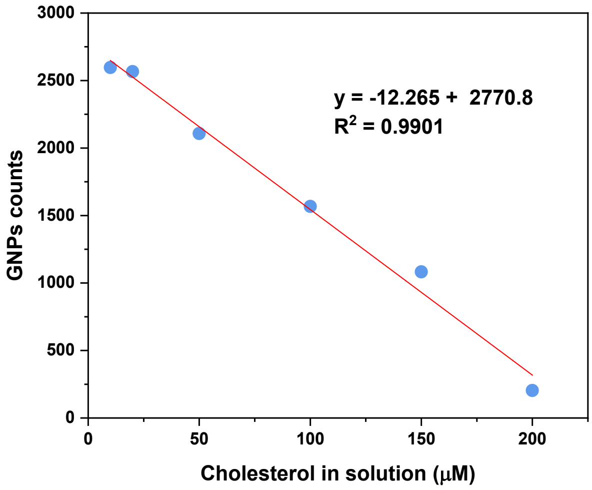

Figure S3. Calibration curve of the competitive bioassay for the system Si-GA-APTES-C-pept for the quantification of free cholesterol in solution (PBST). All data reported are presented as the mean of n ≥ 3.
